# Bio-protective effects of *Lactobacillus plantarum* subsp. *plantarum* against aflatoxin b1 genotoxicity on human blood lymphocytes: a native probiotic strain isolated from Iranian camel milk

**DOI:** 10.18502/cmm.6.4.5438

**Published:** 2020-12

**Authors:** Parvaneh Afshar, Leila Roozbeh Nasiraie, Mohammad Shokrzadeh, Azade Ghorbani HasanSaraei, Shahram Naghizadeh Raeisi

**Affiliations:** 1 Department of Food Science and Technology, Islamic Azad University, Ayatollah Amoli Branch, Amol, Iran; 2 Research and Development Unit of Referral Laboratory, Deputy of Health Management, Mazandaran University of Medical Sciences, Sari, Iran; 3 Department of Food Science and Technology, Islamic Azad University, Nour Branch, Nour, Iran; 4 Department of Research and Development, Shams Bavaran Salamat Nour Company, Tehran, Iran; 5 Department of Toxicology and Pharmacology, Faculty of Pharmacy, Mazandaran University of Medical Sciences, Sari, Iran; 6 Pharmaceutical Sciences Research Center, Faculty of Pharmacy, Mazandaran University of Medical Sciences, Sari, Iran

**Keywords:** Genotoxicity, Health, *Lactobacillus*, Mycotoxins, Probiotics

## Abstract

**Background and Purpose:**

Aflatoxin B1 is one of the main poisonous substances in certain kinds of fungi all over the world. The toxin is a serious health threat to humans and livestock, particularly via DNA damage, and induces multiple cancers. Probiotic agents have confirmed positive beneficial effects in DNA protection against various toxic compounds. In this regard, the present study aimed to investigate the bio-protective effects of a native *Lactobacillus plantarum* subsp. *plantarum*NIMBB003 strain isolated from Iranian one-humped camel milk against AﬂatoxinB1 (AFB1)-induced genotoxicity damage, based on the micronucleus test as a genotoxicity monitoring method.

**Materials and Methods:**

In this study, a human male blood sample was treated and incubated with10^7^, 10^9^, and 10^11^CFU/mL of viable *L. plantarum* and IC50 dose ofAFB1alone and in combination. Afterward, assessed the rate of production of the micronucleus in bi-nucleated lymphocytes. It must be noted that a p-value of less than0.05 was considered significantly significant.

**Results:**

Based on the findings, the combined treatment of the *L. plantarum* at 10^11^ and10^9^CFU/mL dose with 5.33±0.57% of the micronuclei fragments had protective effects and significantly decreased the genotoxicity of AFB1 by 76%.

**Conclusion:**

According to the findings, it can be concluded that *L. plantarum* in 10^9^ CFU/mL had high protective potency against AFB1 genotoxicity. Consequently, the use of local, natural, and native protected compounds with antioxidant effects, such as probiotics agents, is one of the objectives of developing a green strategy in macro-management policies for the discovery and production of new medicines and functional foods with protective/therapeutic effects against nutritional and endogenous DNA toxins.

## Introduction

Removal or decrease of food industry challenges is one of the serious requirements based on specific approaches to the security policies in all countries [ 1 , 2 ]. Chemicals as well as biological and environmental hazards, such as AﬂatoxinB1 (AFB1), pose the most dangerous challenges to mammals [ 3 , 4 ]. The AFB1 is a biological compound that plays a role in the survival of the fungi and has an inconsistent effect on the environment [ 3 , 5 ]. 

The AFB1is a potent carcinogen for humans as well as many animal species, including mice, ﬁsh, and primates. The International Cancer Research Agency classified it as the category 1 human carcinogen [ 6 ]. Main mechanism of this liposoluble compound is the production of free radical species of an unstable and highly reactive toxigenic metabolite group, namely aflatoxin-8, 9-epoxide. This metabolite induces critical damage in vital macromolecules, such as DNA, and changes to more stable molecular cases causing chronic diseases, including cancers, in humans and many animal species that often lead to death [ 7 - 9 ]. The toxicity is related to the host species, gender, and age as well as the ingested dose and the duration of the exposure [ 3 ]. 

The food polluted with AFB1 can be decontaminated through physical and chemical procedures in post-harvest practices. However, these treatments can lead to adverse properties in food products, such as lack of nutrients, quality, and flavor, and also require considerable investment [ 10 ]. Therefore, in recent decades, green policy procedures have been developed to eliminate DNA-reacting compounds, such as AFB1. There are two eco-friendly protection approaches for the elimination of mycotoxins genotoxicity, consisting of herbs, microbes, and their derivatives. Both of these strategies can be applied in individual, combined, or Nano forms [ 10 - 12 ]. 

The bio-procedure suggests numerous species of microorganisms, batteries, yeasts, actinomycetes, molds, and algae that are called probiotics and can reduce aflatoxins in food and feed. Their beneficial properties are associated with their ability to adhere to different targets or bind them [ 13 , 14 ]. The DNA bio-protective effect depends on the probiotic strain, which can effectively display different antigenotoxic or even genotoxic effects. Generally, the antigenotoxic and antimutagenic activities of these probiotics are recognized as a new functional property [ 3 , 10 , 15 ]. 

Probiotics are defined as “living microbial of food products/supplements that, if consumed in adequate amounts as a portion of food, will have beneficial effects on the health of humans and animals” [ 16 , 17 ]. According to the results of previous studies, the microbial exopolysaccharides (EPSs) are primary or secondary metabolites produced by microorganisms. They have been used as prebiotics which have the highest antioxidant activities. Currently, there is considerable interest in the potential antigenotoxic and anti-carcinogenic effects of probiotics [ 18 , 19 ]. *Lactobacillus plantarum* candidate as a probiotic is a Gram-positive and bacilli-shaped organism. Aerotolerant bacteria can grow at temperature and pH rangesof12-40 °C and 3.4-8.2, respectively, and produce the highest amount of EPS [ 18 , 20 ]. 

Due to the side effects of chemical medications, a green strategy has been developed with the approach of using natural antioxidant compounds. The present study aimed to investigate the bio-protective effects of the non-pathogenic native strain *L. plantarum* subsp. *plantarum* NIMBB003 (LPPN) isolated from camel milk against genotoxicity damage induced by AFB1 in cultured human lymphocytes by micronucleus(MN) assay. This ability can be considered as one of the functional properties of the isolates. 

## Materials and Methods

**Chemicals**

Solution 1000 ng/mL of AFB1 (C_17_H_12_O_6_), phytohaemagglutinin-M (PHA), Cytochalasin B, MTT (thiazolyl blue tetrazolium bromide), Amphotericin B, Cisplatin, and Vitamin C were purchased from Sigma Aldrich (St. Louis, MO, USA). RPMI 1640 medium, Fetal bovine serum (FBS), penicillin and streptomycin were manufactured in Gibco (Gibco-BRL, Germany). Moreover, De Mann Rogosa and Sharpe (MRS) agar as well as the broth media were provided by Micromedia (Iran). Human primary gingival fibroblast (HGF) normal cell line was purchased from Pasteur Institute of Iran, Tehran, Iran. All other reagents were purchased based on the standard chemical grade. 

**Microorganism origin, growth media, and cultivation conditions**

The *L. plantarum* strain was obtained from the culture collection center of Cellular laboratory of Shams Bavaran Salamat Nour Company, Tehran, Iran. The strain was a new native probiotic strain isolated from one-humped camel raw milk flora in Golestan province, Iran that was utilized and confirmed by reverse transcription-polymerase chain reaction amplification of 16S rRNA gene sequencing. According to EzTaxon analysis and with the highest levels (100%) similarity of 16s rRNA sequence (1426bp), the strain was determined as *L. plantarum* ATCC 14917. Our strain was recorded with MT012188.1code number as a unique sequence in the NCIB/BLAST database. The selection criterion of the strain was according to the high amount of polysaccharides (particular dextran) of the cell wall (https://www.ncbi.nlm.nih.gov/nuccore/MT012188.1/). 

Stock cultures were maintained at -80˚C in 20% (v/v) glycerol in the Research and Development Unit of Referral Laboratory, Mazandaran University of Medical Sciences, Sari, Iran. The working culture was prepared from frozen stock by transfer in MRS broth and incubation at 37 ± 0.5 °C anaerobic conditions (5% CO_2_) for 11±2 h without agitation based on the isolated growth curve. The bacteria were harvested by centrifugation (1500×g, 10 min, 4˚C) and washed twice with sterile Ringer's solution (pH 7.3). Thereafter, the bacterial density was adjusted to 10^11^ CFU/mL up to 600 nm and the optical density was estimated at 2.1. Afterward, the bacteria with 10^9^ and 10^7^CFU/mL cells were prepared with two-fold serial dilutions 1:100(v/v). 

**Cell line culture**

HGF cell lines were cultured in RPMI-1640 media with 10% fetal bovine serum, 100µg/mL streptomycin, and 100IU/mL penicillin. It should be mentioned that they were maintained at 37°C in a 5% CO_2_ atmosphere. Daily evaluation of cell culture was performed under an inverted microscope and adjusted to allow for ~80% exponential growth. 

**Determine of AFB1 IC_50_ dose via MTT assay**

The protocol was adapted from the described method by Shokrzadehet al. [ 21 ]. The MTT test was used to assess cell viability based on the capacity for viable cells to metabolize a tetrazolium colorless salt to a blue formazan in mitochondria. The HGF monolayer (of previous step) was washed three times with phosphate-buffered saline (PBS) (pH 7.4). Afterward, it was seeded in a 96-well microplate (1 x 106 cells/ml/well) for 24 h with 5% CO_2_ and 95% O2 at 37°C. That time added 50µL/well of AFB1 (0,5,10, and 20 µM) and PBS was used as the negative control. After 24 h of incubation, the media from each well were carefully removed and washed three times with PBS. Subsequently, MTT (20 µL, 5 mg/mL) was added to each well and incubated for four h. The mitoch ondrial NADPH enzyme of viable cell line converted the soluble tetrazolium to insoluble formazan. Afterward, they were washed twice with the PBS solution and was added 100 µl of dimethyl sulfoxide to them for changed to the formazan soluble formed. Subsequently, the wells were shaken for 15 min to extract the formazan formed in the viable cells. Absorbance was determined at 570 nm for each well using a microplate reader. The cell viability was expressed as a percentage of the viable cells with respect to the control and blank group via the Equation:

*Cell viability%=OD Sample-OD Blank × 100 / OD Control*

**Evaluation of *Lactobacillus plantarum* subsp. *plantarum* NIMBB003 isolate and AFB1 co-incubation cell viability**

The LPPN cell viability growth was assessed after co-incubation with the AFB1 in MRS media. A part of the overnight bacterial culture in the previous step was treated with AFB1 IC_50_ dose at 37 °C for 120 min. Afterward, the cells were washed three times in the sterile Ringer's solution and a suitable dilution (10^11^ CFU/mL) was added to the MRS agar media, incubated at 37°C for 18 h. The viability percentage was calculated by the plate count method (CFU/mL). In addition, controls indicate were lactobacilli alone as positive control and the culture media alone and AFB1 alone as the negative control. 

**Donor and blood sample collection**

The heparinized peripheral blood sample was obtained from a healthy male individual within the age range of 25–30 years old who did not consume cigarettes, alcohol, or any known medical and herbal medications. It must be noted that the volunteer was willing to participate in the study and written informed consent was obtained from him. The research was approved by the Ethics Committee of Mazandaran University of Medical Sciences, Sari, Iran (IR.MAZUMS.REC.1398.4715). 

**Lymphocyte cultures and micronucleus assay**

The MN test was conducted in human lymphocytes of whole blood cultures according to the Shokrzadeh et al. [ 22 ] method. Briefly, heparinized whole blood samples (0.5 mL) were added to 4.5 mL of RPMI 1640 medium, supplemented with 20% heat-inactivated fetal bovine serum, 1% antibiotics (penicillin100U/mL, streptomycin100mg/mL, and amphotericin B 100mg/mL), 2% L-glutamine, and 1% PHA as lymphocytes divided stimulate. Finally, they were incubated in a CO_2_ incubator at 37°C for 24 h. 

Subsequently, the cultures were treated with 10 µM of AFB1 and 500 µl/mL of every bacterial serial dilution. Cytochalasin B (Cyt-B) at 6µg/mL concentration was added 44 h after PHA stimulation. Therefore, the Cyt-B inhibition cytokinesis in mitosis phases cusses the appearance of a multi-nuclear cell due to genotoxic compounds. 

According to the previously published criteria [ 21 ], the bi-nucleated lymphocytes were harvested 72 h after the start of incubation by fresh hypotonic KCl (0.075M) treated as red blood cell lysis. Afterward, the sediment was followed by three replicative cycles of fresh methanol-acetic acid (3:1) as a fixation reagent. Subsequently, the fixed cells on sediment were collected and spread onto three glass slides where they were left to be air-dried. Eventually, they were stained with 10% Giemsa for 12 min and assigned blind codes. 

Cells with two macronuclei surrounded by cytoplasm and a cell membrane were scored for the presence of MN. The slides were observed at ×40 and ×100 magnifications using a Nikon light microscope to estimate frequency based on the number of MN in at least 1000 bi-nucleated cells (lymphocytes that were once divided by mitosis) for each case. The negative and positive control cultures were treated based on a previous study with a toxic dose of Cisplatin and a protective dose of Vitamin C concentrations. The experiments were performed on nine different groups and are summarized in Table 1. 

**Table 1:** Description of treatment groups

Number Group	Description
1	Negative Control (without any treatment)
2	Cisplatin (0.750 μg/mL) alone as AFB1 Positive Control
3	AFB1 IC_50_ alone
4	Vitamin C (0.16 µg mL-1) alone as *Lactobacillus plantarum* Positive Control
5	*Lactobacillus plantarum* (10^11^ CFU/ mL) alone
6	AFB1 IC_50_ + *Lactobacillus plantarum* (10^11^ CFU/ mL)
7	AFB1 IC_50_ + *Lactobacillus plantarum* (10^9^ CFU/ mL)
8	AFB1 IC_50_ + *Lactobacillus plantarum* (10^7^ CFU/ mL)
9	AFB1 IC_50_ + Vitamin C (0.16 μg/mL) as Positive Control in treatment

**Statistical analysis**

Statistical analysis of the data was performed in GraphPad Prism software (version 8.3.0). The numbers of MN were recorded for each group. It was found that the data were normally distributed; therefore, all the data were expressed as the mean value of three replicates for each treatment. One-way analyses of variance with Tukey’s honestly significant difference post hoc test were used for multiple comparisons. It must be noted that a p-value of less than 0.05 was considered significantly significant. 

## Results

**Determine of AFB1 IC_50_ dose via MTT assay**

**Figure 1:**
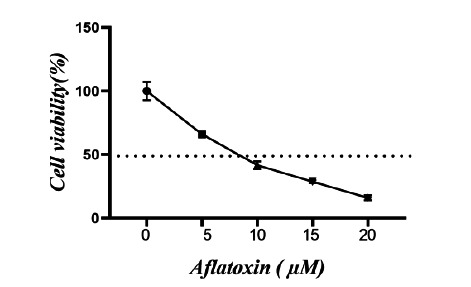
AFB1 IC_50_ dose on the human primary gingival fibroblast cell line

The AFB1 concentration-response curve was established from the data obtained from the HGF cell (Figure 1). Based on the results, AFB1 consistently reduced cell viability in a concentration-dependent dose. After24 h of exposure, the IC_50_ of AFB1 was estimated at 8.99 µM. 

**Viability of *Lactobacillus plantarum* subsp. *plantarum* NIMBB003cells in MRSA media**

**Figure 2:**
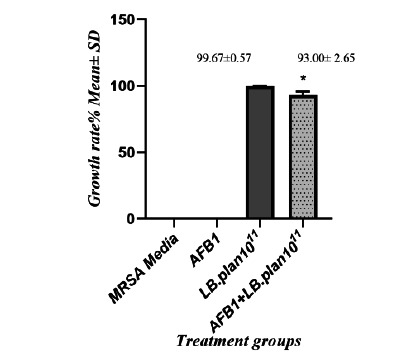
Bacterial cell viability in MRSA

The growth rate and viability of LPPN cells treated with AFB1 in MRSA media was shown in Figure 2. Evaluation result of the viability of LPPN cells treated with AFB1 was within the acceptable growth range of 93.00±2.65% (*P<0.01*). 

**Genotoxicity assay of *Lactobacillus plantarum* subsp. *plantarum* NIMBB003and AFB1alone (pretreatment stage)**

**Figure 3:**
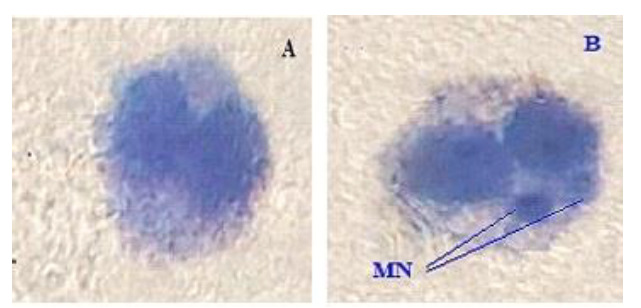
Human lymphocyte cell, A: Normal bi-nucleated cell, B: Abnormal bi-nucleated cell with two micronuclei(MN)

Effects of different doses of the native isolate on MN frequency in bi-nucleated lymphocytes induced of AFB1 are shown in Table 2 and Figures 3. In comparison to lymphocytes MN rate with negative control (0.67±0.57%), LPPN isolate alone in maximum dose (10^11^ CFU/mL) with 1.00±1.00% did not have a significant impact, similar to Vitamin C treatment (1.67±1.53%). In contrast, the MN level in AFB1 alone (22.33±4.51%) with a significant difference increased to 33-foldwhich was almost similar to Cisplatin treatment (33.33±4.17%). 

**Table 2:** Effects of different doses of treatments on peripheral blood lymphocytes micronuclei frequency based on *Lactobacillus plantarum*, Aflatoxin B1, and controls groups (P&lt;0.05)

Groups	1	2	3	4	5	6	7	8	9
	Negative Control	Cisp^a^	AFB1^b^	Vit C^c^	L.P 10^11^ ^d^	AFB1+ L.P10^11^	AFB1+ L.P10^9^ ^e^	AFB1+ L.P10^7^^f^	AFB1+ Vit C
Mean ±SD	0.67±0.57	33.33±4.17	22.33±4.51	1.67±1.53	1.00±1.00	5.33±0.58	5.67±0.58	13.67±1.53	3.00±1.00
Ratio of micronuclei changes	-	50-fold↑^Փ^	33-fold↑^Փ^	2.5-fold↑^Փ^	1.5-fold↑^Փ^	76.1%↓^¶^	74.6%↓^¶^	38.8%↓^¶^	86.6%↓^¶^

a:Cisplatin; b:Aflatoxin B1; c: Vitamin C; d: *Lactobacillus plantarum* 10^11^ CFU/mL; e: *Lactobacillus plantarum* 10^9^ CFU/mL; f: Aflatoxin B1+*Lactobacillus plantarum* 10^7^ CFU/mL; Փ: Compare to Negative control; ¶: Compare to AFB1 group

**Protective role of *Lactobacillus plantarum* subsp. *plantarum* NIMBB003 on AFB1 genotoxicity (treatment stage)**

**Figure 4:**
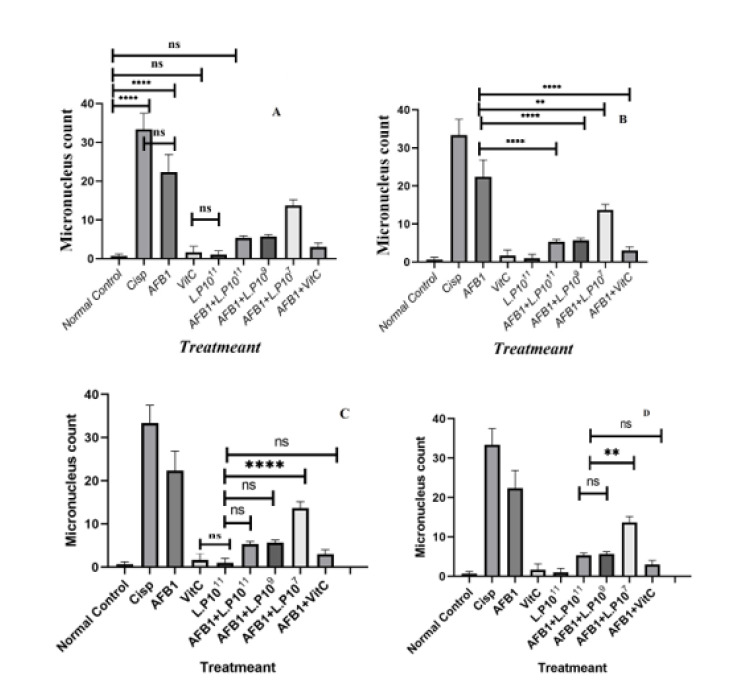
Effects of native Lactobacillus plantarum against Aflatoxin B1 genotoxicity on micronucleus rate of peripheral lymphocytes(A-D). Normal control: without treatment; Cisp: Cisplatin; AFB1: Aflatoxin B1; Vit C: Vitamin C; LP1011: Lactobacillus plantarum 1011 CFU/mL; LP109: Lactobacillus plantarum 109 CFU/mL; LP107: Lactobacillus plantarum 107 CFU/mL; NS: no significant difference (*P>0.05*); *: *P <0.05*; **: *P<0.01*; ***: *P<0.001*; ****: *P<0.0001*.

The protective effect of different doses of the LPPN strain on the MN inhibition rate induced of AFB1 in bi-nucleated lymphocytes are shown in Table 2 and Figures 4-B, C, and D. Comparison of the lymphocytes MN rate of treatment groups with AFB1 group revealed a significant difference of the dose-dependent LPPN on the decrease of MN rate due to AFB1 of 76.1%, 74.6%, and 38.8% in 10^11^, 10^9^, and 10^7^ CFU/mL, respectively. As well, was not observed any significant difference in the two concentrations of 10^9^ and 10^11^ CFU/mL of LPPN. The vitamin C control group was shown a strong protective role with an 86% decrease in the MN rate. 

## Discussion

Different studies have established that lactobacilli can effectively eliminate AFB1 [ 23 , 24 ]. In the present study, we investigated the protective ability of the native LPPN strain against AFB1-induced DNA injuries. This approach is a recent technology that promotes genome health against genotoxicity damage of different foodborne hazards, particularly AFB1, based on the MN assay. 

The MN assay method was performed with the available protocols for whole blood lymphocyte cultures, isolated lymphocytes, and cell lines [ 25 ]. The MN assessment with whole blood lymphocyte cultures seems to be sufficiently sensitive, simple, and fast to detect agents that induce structural and numerical chromosomal aberrations [ 21 , 26 ]. It is known that the genotoxicity of AFB1 involves the induction of oxidative stress and inhibition of different DNA repair mechanisms. The AFB1 interferes with mitochondrial dysfunction through low ATP levels along with increased ROS production and mitochondrial permeability transition [ 14 ]. 

Based on the results, the treatment of human blood lymphocytes with AFB1-induced DNA damage led to a significant 34-fold increase in the MN number. Moreover, the outcome of the Cisplatin treatment in the negative control group was almost the same with a 51-fold increase (Figure 4A). These findings were in line with those of a previous study conducted by Corcuera et al. [ 27 ] which revealed that AFB1 induces a significant increase in the percentage of bone marrow cell MN in rats after 24 h. However, the results of Ochratoxin A(OTA)were negative, albeit both toxins caused toxicity to the bone marrow. 

In the combined treatment, OTA decreased the toxicity and the number of MN produced by AFB1. The toxin type and dose are the main reasons for the induction of oxidative stress and also have a regulatory effect on other toxins [ 14 ]. Based on the findings of another investigation, AFB1 administration to human lymphocyte culture results in increased sister chromatid exchanges as well [ 28 , 29 ]. Peng et al. [ 30 ] and Chen et al. [ 31 ] in their studies found an increased AFB1-induced DNA fragmentation among broilers. According to the results of another study carried out by Rania Jebali et al. [ 32 ] on mice intestinal, AFB1, or aflatoxin M1increased the DNA fragmentation. 

Given the above-mentioned results, this study aimed to assess the native bacterial protective effect against DNA damage, and thereby the treatment with the maximum dose of our isolate (10^11^CFU/mL) required for confirming bacteria safety and viability (Figure 4A). Jebali et al. in their study found no significant changes in the DNA fragmentation of the mice treated with *L. plantarum* alone [ 32 ]. 

Our results showed no significant difference between the micronuclei amounts of vitamin C and negative control groups (Figure 4A). Even in the study conducted by Pizzilitto et al., the bacterial cells led to a slight reduction in the MN rate [ 23 ]. In the present study, the amount of bacterial cell viability was also measured on MRS media and the results revealed the rate of viability to be 92.33±2.86%( Figure 2). This rate was high and consistent with the findings of other published works [ 27 ]. In other words, it was concluded that the native probiotic lacked any genotoxic side effects and had a strong potential benefit for human health. 

Combined treatment of viable native LPPN and AFB1 IC_50_ dose produced a strong protective effect dose depended on human blood lymphocytes genotoxicity (Figure 4B, C, and D). Therefore, treatment 10^7^hada significant difference with10^9^ and 10^11^CFU/mL. Otherwise, there was no significant difference between Vitamin C and bacterial treatment (Figure 4C). Results of the present study are consistent with those of a research performed by Li Huang which indicated the protective effect of viable *L. plantarum* C88 1010 CFU/mL against AFB1 [ 33 ]. Furthermore, Prete reported [ 34 ] that *L. plantarum* could inhibit 75% of the genotoxicity effect produced by 4-NQO using the SOS-Chromotest method. 

Combined treatment of AFB1 with two final doses of viable bacteria 10^9^ and 10^11^CFU/mL did not lead to a significant difference regarding the inhibition or reduction of lymphocyte damage (Figure 4D). As the presence of a suitable amount, at least 108-10^9^CFU of viable cells, in the probiotic formulation is a vital criterion for health claims [ 35 ]. Consequently, based on the results of the present study, it seems that the sufficient dose for prophylactic potential versus AFB1 genotoxicity is 10^9^ CFU, and the increase of bacterial cell to 10^11^ CFU/mL amount or more probably has therapeutic effects. 

The *L. plantarum* spp. is mostly found in fermented foods and exhibits EPS production ability [ 36 ]. The EPS produced by this strain and other LAB strains have severe health effects. The EPSs are among the main components that play a key role in probiotic activities, including anti-genotoxicity and anti-tumor activities, adhesion, and immunomodulation [ 18 ]. Based on previous reports, the EPS of LAB species containing antioxidant activity and non-toxicity, which can be replaced by synthetic antioxidants, are of great importance [ 36 , 37 ]. 

Probiotics can detoxify aflatoxins (AFs) using the biodegradation or bioadsorption mechanisms. The AFs biodegradation occurs when there is a cleavage of the difuran ring of the toxin via probiotic cells and their enzymatic metabolites. In the present study, bioadsorption was performed by specific strains of probiotics organisms, such as *Lactobacillus* bacteria, and using different physical attachment mechanism to the cell wall [ 14 ]. 

Generally, two main mechanisms are recommended for EPS of LAB protection against AFs in both viable cell and their cell lysates mode. First, EPS of LPs has a strong ability to bind with AFs via noncovalent weak interactions, reduced bioavailability of toxins, and increased excretion of fecal AFB1[ 32 , 33 ]. The reason is that the ability to bind and remove AFB1 in various strains is different in order to distinguish their effectiveness [ 32 ]. Second, EPS synthesized by LAB has important therapeutic and physiological qualities, such as the antioxidants and radical scavenger’s properties. Besides, their potential for the elimination of hydrogen peroxide and superoxide consequently leads to the removal of enhanced oxidative stress and DNA-damage induced by the aflatoxins. 

## Conclusion

Concerns about the adverse health effects of AFs have resulted in the investigation of approaches for the reduction, inactivation, or elimination of the bioavailability of the toxin in contaminated products. It seems to have been one of the goals of the green government in global macro policies during the last decades. Furthermore, people have started to pay more attention to the uses of medicines and functional food with antioxidant properties based on traditional natural elements that exist in our surroundings. They are sources of different herbs, microbes, and their derivative products that may promote the health of the consumers. 

Findings of the present study confirmed the genotoxicity induced by aflatoxins B1 in human lymphocytes which was accompanied by a pseudo elevation in MN levels. Moreover, the results demonstrated that the native viable probiotic cell LPPN did not have any adverse effects on genotoxicity and also had a protective role against the mycotoxin gene damage. Therefore, it can be used as a safe alternative food additive to limit food complications severity and improve the beneficial effects. However, further studies are needed to provide a better understanding ofthe in-vivo possible mechanism(s) based on various sections of cells, particularly the EPS cell wall, which may reduce the toxicity induced by the mycotoxin or their not well-known side effects. 

## Authors’ contribution

P.A., M. Sh., and L. R. N. conceived and designed the survey and wrote the paper P. A. and L. R. N. conducted the study and collected and prepared the microbial sample. A. and M. Sh. performed the genotoxicity experiments and the statistical analysis. This work was the result of the collaboration fall authors P. A., M. Sh., L.R. N., A.Gh. H., and Sh. N.R. Moreover, all authors have read and approved the final manuscript.

## Financial disclosure

This study was supported by Mazandaran University of Medical Sciences, Sari, Iran (project number 7515).
